# Myasthenia Gravis Mimicking Status Asthmaticus: The Hidden Crisis

**DOI:** 10.7759/cureus.53044

**Published:** 2024-01-27

**Authors:** Saket Toshniwal, Anil Wanjari, Sourya Acharya, Sunil Kumar, Tushar Sontakke

**Affiliations:** 1 Medicine, Jawaharlal Nehru Medical College, Datta Meghe Institute of Higher Education and Research, Wardha, IND

**Keywords:** myasthenic crisis, non-respiratory cause of dyspnea, subtle signs, status asthmatics, myasthenia gravis

## Abstract

Status asthmaticus is a severe form of aggravation of asthma, whereas myasthenia gravis (MG) is a rare neuromuscular condition characterised by exhaustion and muscle weakness. Myasthenic crisis can occasionally manifest with symptoms that resemble status asthmaticus, which can result in an incorrect diagnosis and ineffective therapy. In addition to discussing the therapeutic implications, this abstract attempts to draw attention to the difficulties in distinguishing between status asthmaticus and myasthenia crisis and the importance of diagnosing subtle signs of MG. In this case, we present a 55-year-old female, with a misdiagnosed case of bronchial asthma, who presented with shortness of breath at rest for two to three days and was suspected to have an acute exacerbating episode of asthma. She was later evaluated for non-respiratory causes of dyspnea on noticing subtle signs of ptosis and was found to have an active myasthenic crisis. Although this case presented typically as status asthmaticus, it did not respond to conventional treatment of it, and on the contrary, it worsened. Hence, it is necessary to look for subtle signs of MG and promptly differentiate it from other similar emergency events to help administer accurate treatment which can prove life-saving.

## Introduction

Myasthenia gravis (MG) is an autoimmune neuromuscular disease that mostly affects the voluntary muscles. It is characterised by fatigue and muscle weakening. The disease has an incidence of between 4.1 and 30 cases per million person-years and a prevalence rate of between 150 and 200 cases per million globally [1]. A neuromuscular junction (NMJ) damaging autoimmune reaction is part of the pathogenesis of MG. Muscle contractions are normally triggered by the binding of acetylcholine, which is produced from nerve terminals to acetylcholine receptors (AChRs). AChRs are blocked, destroyed or otherwise impaired in MG due to the formation of antibodies against the receptors, which reduces the number of available receptors or interferes with their regular operation. This leads to tiredness and muscle weakening as a result of a decreased ability of nerve signals to initiate muscle contractions [2]. Antibodies directed against distinct proteins, such as lipoprotein receptor-related protein 4 (LRP4) or muscle-specific kinase (MuSK), which are also essential for neuromuscular transmission, are involved in another form of MG known as seronegative MG [2]. A serious escalation of MG, known as a myasthenic crisis, is a medical emergency that presents challenging diagnostic and treatment options.

The synthesis and binding of these autoantibodies cause a sharp decrease in functional AChRs at the NMJ during a myasthenic crisis. This causes a major reduction in the nerve impulses that reach the muscle, which causes severe muscular weakness. A myasthenic crisis causes significant impairment to the muscles involved in breathing and swallowing, which can lead to respiratory failure and other potentially fatal problems [3]. Rarely, like in our case, a myasthenic crisis might manifest as status asthmaticus, a severe and potentially fatal asthma exacerbation [4]. There have been reports of such complex autoimmune cases, which present major difficulties in diagnosis and treatment [5-7]. Accurate diagnosis and timely intervention are essential for handling this intricate clinical situation. Although the presentation of a myasthenic crisis with respiratory involvement and that of status asthmaticus are similar, it is important to differentiate between them as a timely intervention with accurate treatment protocol can prove life-saving, like in our case where a possibility of a non-respiratory cause of dyspnea was considered in an earlier diagnosed case of bronchial asthma on noticing a subtle sign of ptosis in this patient. It is important to bring such cases to the literature to help increase awareness among clinicians about considering non-respiratory causes for dyspnea and to carefully look for subtle signs in the physical examination of the patient, which can help diagnose these rare hidden etiologies. 

## Case presentation

A 55-year-old female presented to the casualty with chief complaints of dyspnea on mild exertion for one month that aggravated in the last three days such that she was unable to complete her sentences. On general examination, the patient's condition was poor with a pulse rate of 130 beats per minute, a blood pressure of 150/90 mm of hg, an oxygen saturation of 84% on room air, and a respiratory rate of 24 cycles per minute. On physical examination, no evidence of pallor, icterus, oedema, lymphadenopathy, cyanosis, or clubbing was observed. The patient was conscious and oriented to time place and person. All deep tendon reflexes were preserved with bilateral flexor plantar. The patient also gave a history of difficulty in swallowing for two months, which was suggestive of bulbar weakness. The possibility of Eaton Lambert syndrome was ruled out as the patient did not have any proximal muscle weakness with all reflexes preserved with predominantly bulbar weakness.

Bilateral ronchi with coarse crepitations and wheezing were heard over more than two-thirds of the lung field. The patient had a history of visits to a primary health care set-up since the start of her complaints. She was misdiagnosed with bronchial asthma and was started on conventional treatment for asthma, which includes bronchodilator therapy (tablet Deriphyllin 150 mg twice a day), which she had been taking for a month. The symptoms didn’t improve even after the conventional treatment of asthma and they kept deteriorating to the point where the patient experienced dyspnea at rest and presented to the tertiary care. Owing to the significant past history of diagnosis of bronchial asthma in primary health care set-up and symptoms favouring it, the patient was suspected to have an acute exacerbation of bronchial asthma and was treated with bronchodilators, steroids, and nebulisation with short-acting beta-agonists along with the non-invasive type of positive pressure ventilation. IV magnesium sulphate was also administered during the attack of exacerbation but the patient worsened on the contrary. On monitoring arterial blood gas, severe retention of carbon dioxide was observed as shown in Table 1. 

**Table 1:** Arterial blood gas analysis of the patient suggestive of severe type II respiratory failure

Parameter	Result	Normal range
pH	7.10	7.35 - 7.45
PaCO2 (partial pressure of carbon dioxide)	75 mmHg	35 - 45 mmHg
PaO2 (partial pressure of oxygen)	50 mmHg	75 - 100 mmHg
HCO3- (bicarbonate)	45 mEq/L	22 - 26 mEq/L
SaO2 (arterial oxygen saturation)	78%	95 - 100%

**Figure 1:**
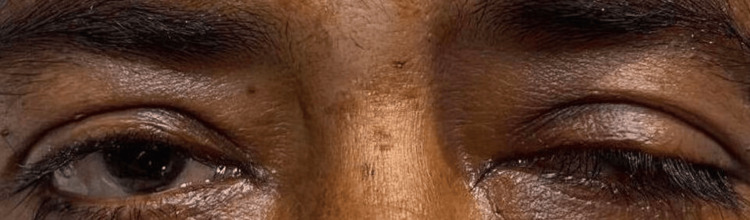
Ptosis of both eyelids prior to ice pack test (left more than right)

The patient was hence electively intubated to treat type II respiratory failure. It was further noticed that the patient had droopy eyelids bilaterally suggesting ptosis (more on the left than right), which is a subtle sign of MG, which was overlooked on admission, as shown in Figure 1. 

**Figure 2:**
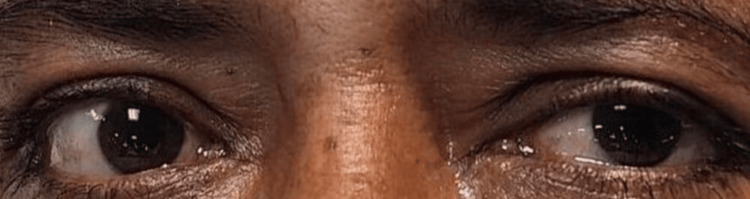
Improvement of more than 2 mm in ptosis of both eyelids post ice pack test

A bedside ice pack test was performed to rule out MG. An improvement in the ptosis of more than 2 mm in both the eyelids was observed as shown in Figure 2.

An AChR antibody titre was tested which came out to be significantly raised (greater than 0.4 nmol/L with a normal range of less than 0.05 nmol/L) and the diagnosis of MG was confirmed. A tensilon test (edrophonium test) was also performed by administering 2 mg IV edrophonium in incremental doses up to 8 mg following which the ptosis of the patient showed improvement, which helped diagnose an active myasthenic crisis. CT chest was done and was found to be normal ruling out the presence of thymoma. While the presence of AChR antibodies can offer insights into the potential presence of thymoma in MG patients, it is essential to consider other clinical and diagnostic factors for a comprehensive evaluation. Single fibre electromyography was performed, which revealed increased jitter in nerve impulses, increased blocking of electrical impulses, and progressive decrease in the amplitude of muscle response during repetitive nerve stimulation suggestive of decremental response, which helped diagnose MG. A diagnosis of a myasthenic crisis was made, which was earlier misdiagnosed as status asthmaticus. The patient was started on IV immunoglobulin therapy (2 gm/kg administered in divided doses over five days) along with anticholinesterase inhibitors (tablet pyridostigmine 60 mg thrice a day) and immunosuppressants (tablet azathioprine 25 mg twice a day). The patient responded well to the treatment and the ventilator was eventually weaned off. The patient improved during the hospital stay and was discharged on tablet azathioprine 25 mg twice a day along with tablet pyridostigmine 30 mg twice a day and was advised regular follow-up.

## Discussion

A myasthenic crisis and status asthmaticus are two distinct medical emergencies that can present with similar respiratory symptoms, leading to potential misdiagnosis and delayed treatment. A myasthenic crisis is a complication of MG, where severe muscle weakness affects the respiratory muscles, leading to respiratory failure. On the other hand, status asthmaticus is a severe and prolonged asthma attack that does not directly involve muscle weakness [8]. In some cases, myasthenic crises can masquerade as status asthmaticus due to overlapping symptoms such as shortness of breath, wheezing, and difficulty in breathing. The weakness of respiratory muscles in a myasthenic crisis can mimic the bronchospasm seen in status asthmaticus. This can lead to misdiagnosis and inappropriate treatment with bronchodilators, which could worsen the myasthenic crisis, like in our case where the patient worsened after IV magnesium sulphate administration to treat the acute episode of status asthmaticus [8]. A misdiagnosis of MG as status asthmaticus can occur due to the similarity of symptoms and the rarity of MG. Many patients with MG experience delays in receiving an accurate diagnosis, leading to prolonged suffering and potential complications. A specific clinical prediction scale for myasthenic crisis as a cause of imminent ventilatory failure is not mentioned specifically in the literature. Nonetheless, the data implies that variables including serum bicarbonate levels, acute physiology and chronic health evaluation (APACHE) II score, and hypercapnia may function as indicators of whether non-invasive ventilation (NIV) is effective or unsuccessful in treating a myasthenic crisis. 

The challenges in identifying MG in patients with dyspnea lie in the need for a high index of suspicion, as well as the reliance on diagnostic tests to confirm the condition. Additionally, the overlap of symptoms between MG and other respiratory conditions, such as asthma, can further complicate the diagnostic process [9]. Differentiating between the two conditions requires a thorough clinical evaluation, including a detailed medical history, physical examination, and diagnostic tests. Key differentiating features include the presence of other typical symptoms of MG, such as ptosis (drooping eyelids), diplopia (double vision), and generalised muscle weakness [3,9]. Several diagnostic tests can help confirm the presence of MG. The first-line test is the AChR antibody test, which detects the presence of antibodies that block or destroy AChRs. A positive AChR test is indicative of MG, but a negative result does not rule out the condition. Additional tests, such as the ice pack test, the repetitive nerve stimulation test, single-fibre electromyography, and the edrophonium (Tensilon) test, may be required to help diagnose myasthenic crisis in cases where the initial tests are inconclusive [10]. The treatment of MG aims to improve muscle strength and reduce symptoms. The primary treatment approach involves the use of medications that enhance neuromuscular transmission, such as anticholinesterase inhibitors (e.g., pyridostigmine) and immunosuppressive agents (e.g., corticosteroids, azathioprine). The management strategies for myasthenia crisis and status asthmaticus differ significantly. While status asthmaticus is primarily treated with bronchodilators, corticosteroids, and supportive measures, a myasthenic crisis requires prompt respiratory support, such as mechanical ventilation, along with interventions targeting the underlying autoimmune process, including IV immunoglobulin (IVIg) or plasmapheresis. Surgical interventions, such as thymectomy, may be considered in certain cases, particularly if the thymus gland is enlarged or cancerous [11].

## Conclusions

MG mimicking status asthmaticus is a rare but challenging clinical scenario. It highlights the importance of considering non-respiratory causes of dyspnea as alternative diagnoses, especially when respiratory symptoms are not responding as expected to conventional asthma treatments. MG when presents with crisis invariably involves the diaphragm and other respiratory muscles. A comprehensive evaluation, including a detailed history, physical examination to look for subtle signs like ptosis and paresis, and paradoxical breathing patterns are not to be missed. Moreover, appropriate diagnostic tests like the bedside ice pack test in the presence of ptosis, AChR antibody test, and Tensilon test are crucial to accurately identify the hidden crisis. Also, they help manage these distinct medical emergencies with accurate treatment and hence improve patient outcomes. Healthcare professionals should maintain a high index of suspicion for MG in patients with respiratory distress to prevent misdiagnosis and ensure timely intervention.
